# High-Order Derivative Detection for FSK Ambient Backscatter Communications in Edge-Intelligent Sensing Systems

**DOI:** 10.3390/s26185733

**Published:** 2026-09-09

**Authors:** Jingjing Wu, Peng Wei, Sa Xiao, Jianquan Wang, Wanbin Tang

**Affiliations:** National Key Laboratory of Wireless Communications, University of Electronic Science and Technology of China, Chengdu 611731, China; 202212220608@std.uestc.edu.cn (J.W.); xiaosa@uestc.edu.cn (S.X.); jqwang@uestc.edu.cn (J.W.); wbtang@uestc.edu.cn (W.T.)

**Keywords:** ambient backscatter communication, FSK, high-order derivative, signal detection

## Abstract

Edge-intelligent sensing systems demand ultra-low-power wireless connectivity to sustainably support massive sensor deployments. Ambient backscatter communication (AmBC) meets this demand by harvesting and modulating existing radio-frequency (RF) signals, eliminating dedicated carriers. However, conventional on–off keying (OOK) demodulation in AmBC is highly susceptible to noise, while existing frequency-shift keying (FSK) alternatives relying on first-order derivatives perform poorly at low signal-to-noise ratios (SNRs), compromising the reliability of edge sensing data. In this paper, we propose a signal detection method that exploits high-order derivatives to enhance the demodulation of FSK-modulated ambient backscatter signals. By analytically evaluating the power of interference and noise after high-order differentiation, we reveal that the interference power is minimized at the second order while the noise power increases monotonically with the derivative order, leading to a favorable trade-off in typical AmBC regimes where the modulation frequency is much smaller than the sampling rate and comparable to the ambient signal bandwidth. We then design a phase-preserving frequency amplitude comparison detection (FACD) rule to recover the embedded information. Simulation results show that the proposed second-order derivative-based FACD achieves the lowest bit error rate among all compared schemes, particularly at low SNR.

## 1. Introduction

The rapid expansion of the Internet of Things (IoT) and edge-intelligent sensing is driving massive deployment of low-power sensors in smart cities, industry, and healthcare [1]. A key bottleneck is the energy consumed by wireless transmission: conventional radio transceivers often consume orders of magnitude more power than the sensing elements themselves, making battery-free or energy-harvesting operation essential for scalable, sustainable edge intelligence [2]. Ambient backscatter communication (AmBC) has emerged as a transformative ultra-low-power solution. Unlike conventional backscatter systems that require dedicated carrier generators—such as those in radio-frequency identification (RFID)—AmBC allows passive terminals to opportunistically harvest and modulate ambient radio-frequency (RF) signals already pervasive in daily environments, including television, cellular, Wi-Fi, FM, LoRa, and Bluetooth emissions [3]. This approach not only eliminates reliance on dedicated carriers, drastically reducing hardware complexity and infrastructure cost, but also enables battery-free operation at microwatt-level power consumption [3,4,5]. The pioneering work by Liu et al. [4] first demonstrated this concept, showing that two battery-free devices could communicate by backscattering ambient TV and cellular signals, achieving 1 kbps over distances of 2.5 feet outdoors and 1.5 feet indoors. Subsequent research has confirmed that AmBC is realized by dynamically controlling the antenna impedance at the backscatter device (BD): toggling the load impedance alters the reflection coefficient, thereby embedding data onto the reflected waveform at negligible power [6,7]. While the broader goal is to support edge-intelligent sensing, this paper concentrates on the communication link reliability that underpins such systems. We emphasize that the present work is a physical-layer signal detection study and does not introduce an edge-intelligence architecture, learning mechanism, or sensing functionality. The proposed detector is designed to improve the reliability of data transmission, which is a prerequisite for any higher-level sensing or decision-making tasks.

To modulate information onto ambient RF carriers, on–off keying (OOK) has become the most widely adopted solution due to its simplicity and ultra-low power consumption. In OOK-based AmBC, the tag alternates between two impedance states, reflecting the ambient signal to denote a binary “1” and absorbing it to denote a “0”, so that the receiver can detect the presence or absence of reflected energy [8,9]. This approach has been validated across a variety of ambient carriers. For example, FM backscatter systems achieve communication ranges of 5–60 feet at 11.07 μW power consumption [10], while LoRa backscatter systems support data rates from 39.5 to 199.4 kbps over distances of 50–200 m [11]. Similarly, Wi-Fi backscatter has been demonstrated by modulating existing Wi-Fi traffic and using standard receivers to detect signal-strength variations [12,13]. Ambient backscatter over cellular signals has also been explored for device-to-device IoT communication [14,15].

When the ambient waveform is known a priori, codeword translation can enhance compatibility with commodity receivers. Pioneered in the HitchHike system [16], codeword translation maps an original transmitted codeword into another valid codeword from the same codebook by manipulating phase, amplitude, or frequency, allowing standard Wi-Fi receivers to decode the backscattered packet without modification [16,17]. This technique was later extended by FreeRider [18] to cover Wi-Fi, ZigBee, Bluetooth, and LoRa, achieving data rates up to tens of Mbps and communication distances up to hundreds of meters, validating seamless integration with legacy infrastructure [16,17,18].

Despite its widespread adoption, OOK (including codeword-translation variants) suffers from fundamental limitations. Energy-detection and phase-jump-detection mechanisms are highly susceptible to noise and interference, particularly in dynamic RF environments [19,20]. In practice, the backscattered signal at the receiver is often orders of magnitude weaker than the direct-path ambient signal, making reliable extraction of subtle amplitude or phase changes extremely difficult [4]. Furthermore, unknown ambient signal characteristics or multiple coexisting RF sources limit the flexibility of commercial devices, leading to reduced range and elevated bit error rates (BERs) [21,22]. Strong direct-link interference (DLI) is a major obstacle, as the receiver must isolate the weak backscatter component from the powerful direct carrier and reflected signal [23,24]. Although successive interference cancellation, multi-antenna beamforming, and frequency shifting have been proposed to mitigate DLI, they often require complex receivers, extra spectral resources, or accurate channel state information, which are impractical for low-cost IoT nodes [25,26,27].

Frequency-shift keying (FSK) has attracted increasing attention as an alternative to OOK because it encodes information via deliberate frequency shifts, providing inherent resilience to amplitude fading and impulsive interference [28,29]. Recent studies have validated FSK-based AmBC across various carriers. For example, Tao et al. [30] showed that MFSK can outperform OOK in terms of BER under multipath fading. In LTE networks, Liao et al. [15] proposed an in-band FSK scheme that exploits cell-specific reference signals to create a Doppler-like shift, separating the backscatter path from the direct path in the frequency domain and thus circumventing DLI [14,15]. More recently, the FM Rider system demonstrated over-100-m communication using binary FSK on ambient FM signals [31].

Nevertheless, traditional FSK demodulation faces significant challenges in ambient backscatter scenarios. Conventional FSK receivers are designed for dedicated, stable carrier sources and are ill-suited for unknown, time-varying, or wideband ambient RF signals [32,33]. Moreover, classical FSK demodulation typically requires the frequency shift to exceed the signal bandwidth to avoid spectral overlap; otherwise, spectral aliasing makes the backscatter signal indistinguishable from the ambient carrier, rendering conventional discriminators ineffective [34]. Existing FSK demodulation schemes for AmBC often rely on the first-order derivative of the received signal to recover frequency information [28]. Although computationally lightweight, first-order derivative detection deteriorates sharply at low signal-to-noise ratios (SNRs), where the weak backscatter component is severely corrupted by noise and interference [35,36]. Experimental evidence shows that first-order derivative methods suffer unacceptably high error rates when the SNR falls below practical thresholds, limiting communication range and reliability [15,22].

Several existing works have attempted to improve FSK demodulation in AmBC using first-order derivatives [28]. While computationally simple, these methods treat the derivative order as fixed and do not investigate whether higher orders can improve performance or what the optimal order should be. Moreover, they lack a theoretical framework to quantify the trade-off between signal enhancement and noise/interference amplification as the derivative order increases. Other advanced FSK demodulation techniques, such as time-frequency analysis, cyclostationary feature detection, and machine learning-based classification, require high computational complexity, accurate prior knowledge of ambient signal statistics, or extensive training data, making them unsuitable for resource-constrained, battery-free IoT devices. Consequently, a systematic study of high-order derivatives for FSK-AmBC that identifies the optimal order and provides an interpretable, low-complexity detection rule remains lacking.

To fill this gap, this paper proposes a high-order derivative-based signal detection method for FSK-modulated AmBC. Our main contributions are as follows.

- We analytically characterize the power of direct-link interference and channel noise after high-order differentiation. The analysis proves that the interference power attains its minimum at the second-order derivative, whereas the noise power increases monotonically with the derivative order. This establishes the second-order derivative as the optimal choice under the typical AmBC regime where the modulation frequency is much smaller than the sampling rate and comparable to the ambient signal bandwidth.
- Based on this insight, we propose a phase-preserving frequency amplitude comparison detection (FACD) scheme that exploits the conjugate symmetry of the frequency-domain signal for reliable information recovery.
- Through extensive simulations, we demonstrate that the proposed second-order derivative-based FACD method outperforms conventional first-order approaches and several baseline detectors in terms of BER, especially in low-SNR conditions.

We note that the term “edge-intelligent sensing” in the title and abstract refers to a broader application context of the proposed communication scheme, i.e., enabling reliable, ultra-low-power data transmission for distributed sensor networks. The performance metric evaluated in this work is the BER of the communication link, which directly governs the fidelity of sensed data delivery. The sensing functionality itself (e.g., environmental parameter estimation or target detection) is beyond the scope of this paper but relies on the communication reliability established herein.

The remainder of this paper is organized as follows. Section 2 describes the system model of the FSK-modulated AmBC system, including the received signal formulation and its spectral characteristics. Section 3 presents the proposed high-order derivative-based signal detection method and the FACD rule. Section 4 provides a theoretical performance analysis, investigating the impact of the derivative order on the signal, interference, and noise components, and examines the computational complexity. Section 5 presents numerical simulation results comparing the proposed scheme with several baseline methods. Finally, Section 6 concludes the paper.

## 2. FSK-Modulated AmBC System Model

**Figure 1:**
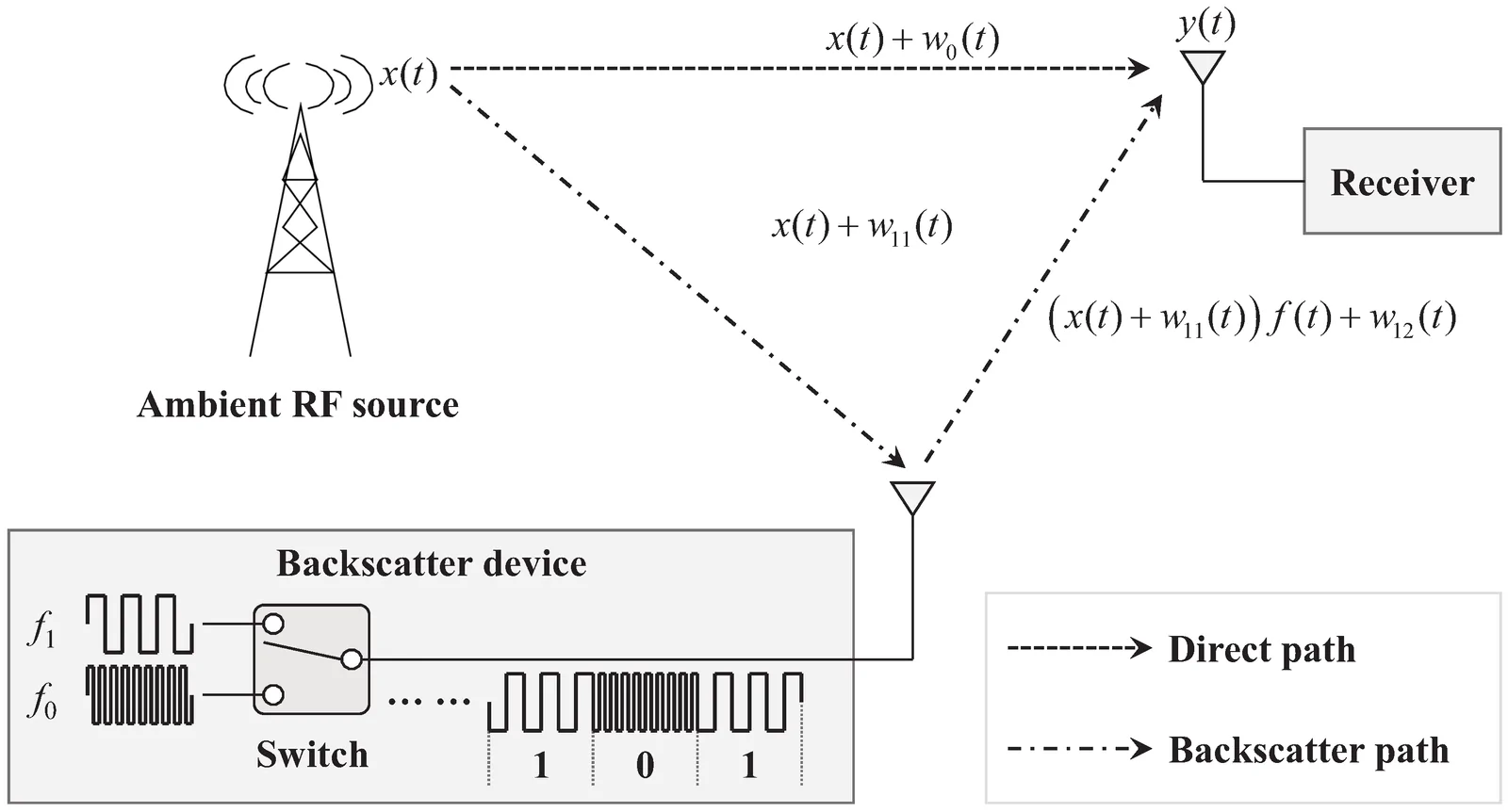
Schematic of the ambient backscatter communication system model.

As shown in Figure 1, we consider an FSK-modulated AmBC system consisting of one ambient RF source, one backscatter device (BD), and one receiver. The ambient RF source continuously transmits an RF signal, denoted by x(t). This signal reaches the receiver via two propagation paths: (1) the direct path from the RF source to the receiver, and (2) the backscatter path from the RF source to the BD and then to the receiver.

This paper establishes a theoretical baseline for high-order derivative detection in FSK-modulated AmBC. To isolate the core signal processing gain and ensure analytical tractability, we adopt the standard assumptions commonly used in initial AmBC studies [28,30,37]: the wireless channels are modeled as additive white Gaussian noise (AWGN), propagation delays are negligible, and perfect synchronization is maintained at the receiver. All noise processes in this paper are assumed to be band-limited by the receiver front-end filter with an effective bandwidth *B*. In addition, the BD is assumed to be placed close to the RF source so that the direct-path and backscatter-path delays are so small that they can be ignored [28]. These simplifications allow us to clearly expose the fundamental advantages of the second-order derivative without being obscured by practical impairments.

Along the direct path, the signal received at the receiver is $x\left(t\right)+w_{0}\left(t\right)$, where $w_{0}\left(t\right)$ denotes the additive noise of the RF source–receiver link.

In the backscatter path, the RF signal first reaches the BD, giving the received signal $x\left(t\right)+w_{11}\left(t\right)$, where $w_{11}\left(t\right)$ is the noise of the RF source–BD link. The BD then modulates its information bits onto the incident RF signal by switching its load impedance between two states in an FSK manner. Denoting the FSK modulation waveform by f(t), the modulated signal is expressed as $(x\left(t\right)+w_{11}\left(t\right))f\left(t\right)$. Specifically, we focus on binary FSK (BFSK), as depicted in Figure 1, where the two switching frequencies $f_{0}$ and $f_{1}$ correspond to information bits “0” and “1”, respectively. Finally, this backscatter signal propagates to the receiver through the BD–receiver link, yielding $\left(x\left(t\right)+w_{11}\left(t\right)\right)f\left(t\right)+w_{12}\left(t\right)$, with $w_{12}\left(t\right)$ being the noise of that link.

Consequently, the receiver captures the superposition of the signals arriving from the two paths. As in [28], we assume that the BD is located close to the RF source, so that the propagation delays of both the direct and backscatter paths are negligible, and perfect synchronization is maintained at the receiver. Under these assumptions, the received signal y(t) can be written as

(1) $$\begin{array}{cc} y(t) & =f\left(t\right)x\left(t\right)+x\left(t\right)+w_{11}\left(t\right)f\left(t\right)+w_{0}\left(t\right)+w_{12}\left(t\right)\\ \; & =f(t)x(t)+x(t)+w(t). \end{array}$$

It should be noted that the lumped noise term $w\left(t\right)=w_{11}\left(t\right)f\left(t\right)+w_{0}\left(t\right)+w_{12}\left(t\right)$ in (1) is introduced only for notational brevity. Strictly speaking, $w_{11}\left(t\right)f\left(t\right)$ is not white Gaussian noise, since multiplication by the FSK waveform f(t) produces a cyclostationary process with spectral peaks at multiples of the switching frequencies. Nevertheless, because the designed detector will evaluate the Fourier transform only at the specific frequencies $f_{0}/2$ and $f_{1}/2$, and because the decision statistic averages over many switching periods, the contribution of $w_{11}\left(t\right)f\left(t\right)$ at these frequencies can be well approximated as equivalent Gaussian noise with finite effective power. This approximation is sufficient for the relative performance analysis of different derivative orders in Section 4.

The target of the receiver is to recover the information modulated onto f(t) from y(t). In (1), the first term f(t)x(t) carries the desired backscatter signal, the second term x(t) represents the DLI, and the third term w(t) is the overall channel noise.

**Figure 2:**
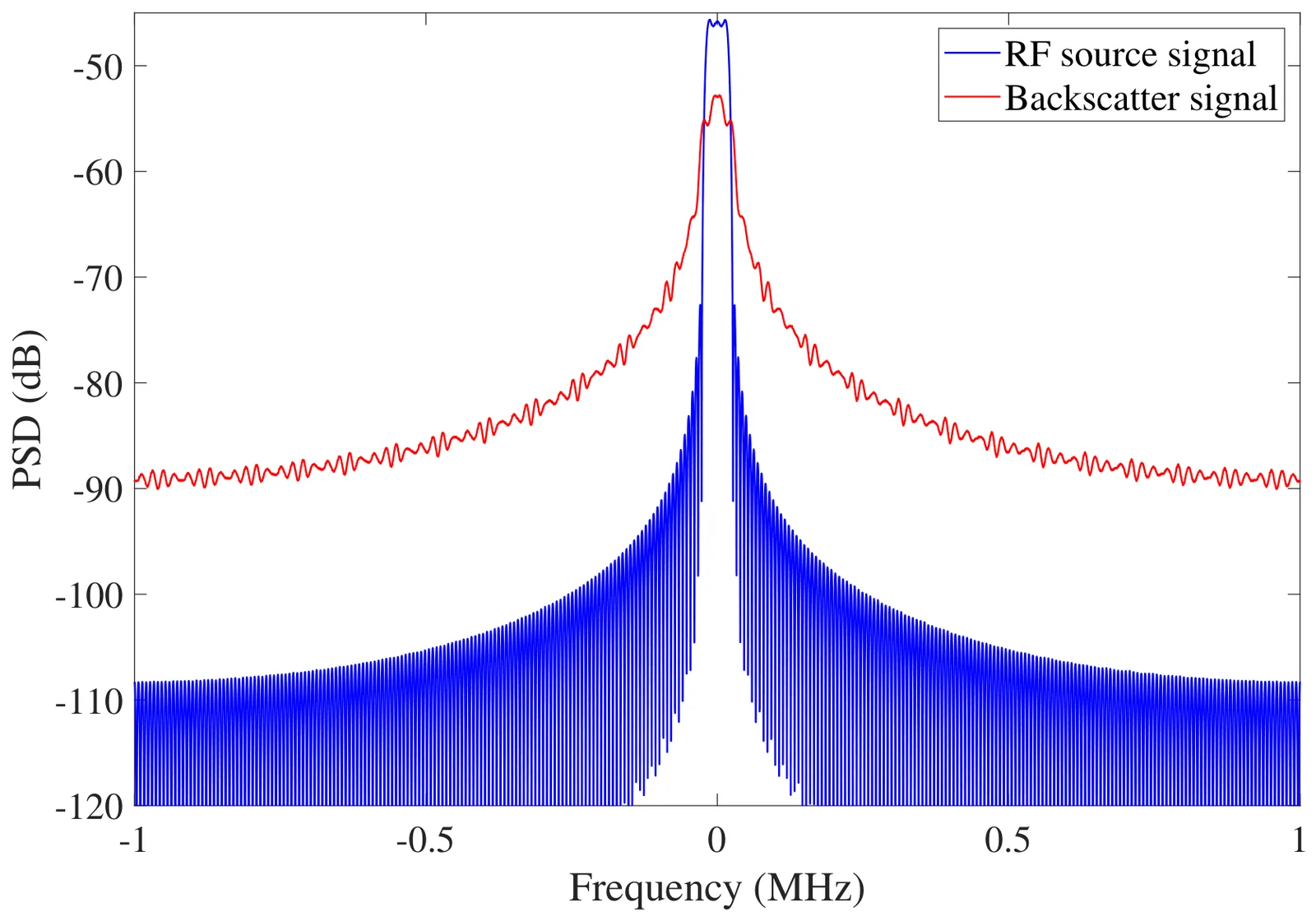
PSD of the backscatter signal.

To facilitate reliable information extraction, it is instructive to examine the spectral characteristics of the backscatter signal. Figure 2 compares the power spectral density (PSD) of the original RF source signal and the backscatter signal, where the simulation parameters are detailed in Section 5. For a bandwidth-limited random RF source, FSK modulation causes a slight bandwidth expansion and a significant out-of-band spectral growth. More importantly, several periodic spectral peaks appear in the high-frequency region, which motivates the signal detection method developed in the next section.

## 3. High-Order Derivative-Enhanced Signal Detection Method

Since f(t) employs FSK modulation, recovering the information bit requires extracting the corresponding frequency $f_{i}$. The differentiation property of the Fourier transform naturally amplifies high-frequency components while suppressing low-frequency ones, which motivates the use of signal differentiation for frequency extraction. In practice, continuous-time derivatives are approximated in the discrete-time domain by finite differences: for a sampled sequence y[k] at a sampling rate $f_{s}$, the first-order difference is Δy[k]=y[k]−y[k−1], and higher-order derivatives are obtained by repeated differencing. The frequency response of the *n*-th order finite difference is ${\left(1-e^{-j2\pi fT_{s}}\right)}^{n}$ with $T_{s}=1/f_{s}$, which approaches ${\left(j2\pi f\right)}^{n}$ for $f\ll f_{s}$, ensuring that the continuous-domain analysis remains valid under the Nyquist sampling condition.

In contrast to conventional first-order differentiation methods [28], this paper adopts a high-order differentiation strategy. The *n*th-order derivative of the received signal y(t) in (1) is given by

(2) y(n)(t)=∑k=0nnkf(n−k)(t)x(k)(t)+x(n)(t)+w(n)(t)=f(n)(t)x(t)+∑k=1nnkf(n−k)(t)x(k)(t)+x(n)(t)+w(n)(t),

where n≥2 and nk=n!k!(n−k)! denotes the binomial coefficient.

In the following, we analyze the characteristics of $f^{\left(n\right)}\left(t\right)$ followed by the proposed signal detection method.

### 3.1. Characteristics of High-Order Derivative of the FSK-Modulated Waveform

**Figure 3:**
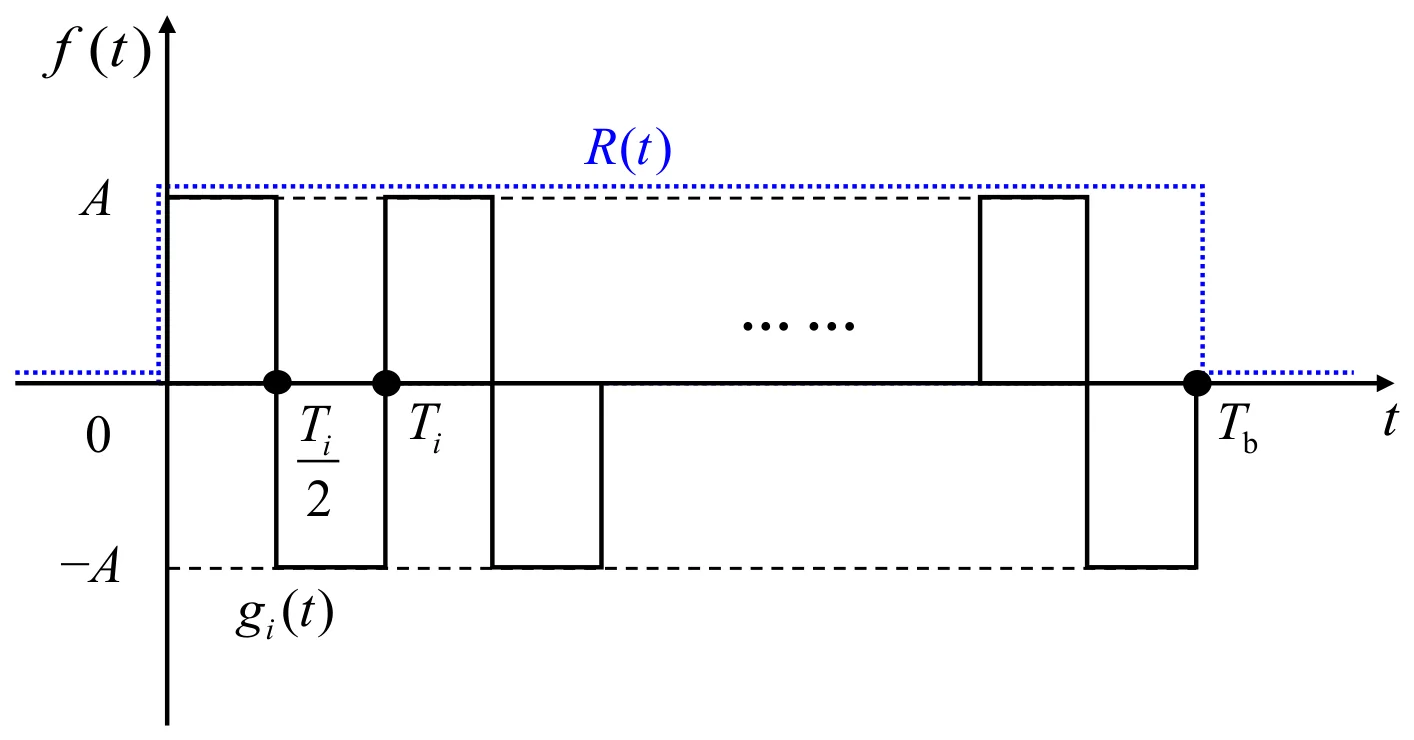
A schematic diagram of the FSK-based modulation waveform.

Recovering the information from the high-order derivative $y^{\left(n\right)}\left(t\right)$ primarily relies on $f^{\left(n\right)}\left(t\right)$, i.e., the first term on the right-hand side of (2). As illustrated in Figure 3, the modulation waveform at the BD is defined as

(3) $$f\left(t\right)=g_{i}\left(t\right)R\left(t\right),\;t\geq 0,$$

where R(t) is the rectangular window function given by

(4) $$R\left(t\right)=\left\{\begin{array}{cc} 1, & 0\leq t\leq T_{b},\\ 0, & \mathrm{otherwise}, \end{array}\right.$$

and $g_{i}\left(t\right)$ is a square-wave signal for FSK modulation expressed as

(5) $$g_{i}\left(t\right)=\left\{\begin{array}{cc} A, & n_{i}T_{i}\leq t<\left(n_{i}+\frac{1}{2}\right)T_{i},\\ -A, & \left(n_{i}+\frac{1}{2}\right)T_{i}\leq t<\left(n_{i}+1\right)T_{i}, \end{array}\right.$$

where $T_{i}$ is the period of the square-wave signal, i=0,1, $g_{1}\left(t\right)$ corresponds to information bit “1”, $g_{0}\left(t\right)$ corresponds to information bit “0”, *A* is the amplitude of the square-wave signal, $n_{i}=0,1,\ldots ,N_{i}-1$, and $N_{i}=T_{b}/T_{i}$ denotes the number of square-wave periods within one bit duration.

Taking the *n*th-order derivative of f(t) in (3) for n≥1 yields

(6) f(n)(t)=gi(n)(t)R(t)+∑k=1nnkgi(n−k)(t)δ(k−1)(t)−δ(k−1)(t−Tb).

**Proof.**  We proceed by mathematical induction. For n=1, we have

(7) $$\begin{array}{cc} f^{\prime }\left(t\right) & =g_{i}^{\prime }\left(t\right)R\left(t\right)+g_{i}\left(t\right)R^{\prime }\left(t\right)\\ \; & =g_{i}^{\prime }\left(t\right)R\left(t\right)+g_{i}\left(t\right)\left(\delta \left(t\right)-\delta \left(t-T_{b}\right)\right). \end{array}$$

Assume that the statement holds for n=l, i.e.,

(8) f(l)(t)=gi(l)(t)R(t)+∑k=1llkgi(l−k)(t)δ(k−1)(t)−δ(k−1)(t−Tb).

For n=l+1, taking the first-order derivative of (8) gives

(9) f(l+1)(t)=gi(l+1)(t)R(t)+gi(l)(t)δ(t)−δ(t−Tb)+∑k=1llkgi(l−k+1)(t)δ(k−1)(t)−δ(k−1)(t−Tb)+gi(l−k)(t)δ(k)(t)−δ(k)(t−Tb).

All terms involving $\delta^{\left(k\right)}\left(t\right)-\delta^{\left(k\right)}\left(t-T_{b}\right)$ in (9) are now collected. The last two sums in (9) can be rewritten as

(10) ∑k=1llkgi(l−k+1)(t)δ(k−1)(t)−δ(k−1)(t−Tb)=∑m=0l−1lm+1gi(l−m)(t)δ(m)(t)−δ(m)(t−Tb),

and

(11) ∑k=1llkgi(l−k)(t)δ(k)(t)−δ(k)(t−Tb)=∑m=1llmgi(l−m)(t)δ(m)(t)−δ(m)(t−Tb).

Let $c_{m}$ denote the coefficient of $\delta^{\left(m\right)}\left(t\right)-\delta^{\left(m\right)}\left(t-T_{b}\right)$ contributed by the second term in (9), (10), and (11). For m=0, the contributions are 1, l1=l, and 0, respectively, yielding

(12) c0=1+l=l+11.

For 1≤m≤l−1, the contributions are 0, lm+1, and lm, which, using the identity lm+1+lm=l+1m+1, gives

(13) cm=lm+1+lm=l+1m+1.

For m=l, the contributions are 0, 0, and ll=1. Hence, we have

(14) cl=1=l+1l+1.

Combining (12)–(14), the last two terms in (9) can be expressed as

(15) gi(l)(t)δ(t)−δ(t−Tb)+∑k=1llkgi(l−k+1)(t)δ(k−1)(t)−δ(k−1)(t−Tb)+gi(l−k)(t)δ(k)(t)−δ(k)(t−Tb)=∑m=0ll+1m+1gi(l−m)(t)δ(m)(t)−δ(m)(t−Tb)=k=m+1∑k=1l+1l+1kgi(l+1−k)(t)δ(k−1)(t)−δ(k−1)(t−Tb).

Therefore, $f^{\left(l+1\right)}\left(t\right)$ becomes

(16) f(l+1)(t)=gi(l+1)(t)R(t)+∑k=1l+1l+1kgi(l+1−k)(t)δ(k−1)(t)−δ(k−1)(t−Tb).

By induction, (7), (8), and (16) establish (6). □

The Fourier coefficients of the square-wave signal $g_{i}\left(t\right)$ are

(17) $$\begin{array}{cc} F_{l} & =\frac{1}{T_{b}}\int_{0}^{T_{b}}g_{i}\left(t\right)e^{-j2\pi f_{l}t}\;dt\\ \; & =\frac{1}{N_{i}T_{i}}\sum_{n_{i}=0}^{N_{i}-1}\int_{n_{i}T_{i}}^{\left(n_{i}+1\right)T_{i}}g_{i}\left(t\right)e^{-j2\pi f_{l}t}\;dt\\ \; & =\frac{A}{N_{i}T_{i}}\sum_{n_{i}=0}^{N_{i}-1}\left(\frac{e^{-j2\pi f_{l}t}}{-j2\pi f_{l}}{|}_{n_{i}T_{i}}^{(n_{i}+\frac{1}{2})T_{i}}-\frac{e^{-j2\pi f_{l}t}}{-j2\pi f_{l}}{|}_{(n_{i}+\frac{1}{2})T_{i}}^{\left(n_{i}+1\right)T_{i}}\right)\\ \; & =A\frac{1-e^{-j\pi l}}{j\pi l}. \end{array}$$

In (17), for even l≠0, $F_{l}=0$; and for odd *l*, $F_{l}=\frac{2A}{j\pi l}$. For l=0, $F_{0}=\frac{1}{T_{b}}\int_{0}^{T_{b}}g_{i}\left(t\right)dt=0$. Let l=2m+1, the Fourier series expansion of $g_{i}\left(t\right)$ is

(18) $$g_{i}\left(t\right)=\sum_{l=-\infty }^{\infty }F_{l}e^{j2\pi f_{l}t}=2A\sum_{m=-\infty }^{\infty }\frac{e^{j2\pi f_{i}\left(2m+1\right)t}}{j\pi (2m+1)}.$$

For n≥1, substituting (18) into (6) yields

(19) f(n)(t)=4A(j2π)n−1finR(t)∑m=−∞∞(2m+1)n−1ej2πfi(2m+1)t+∑k=1nnkδ(k−1)(t)−δ(k−1)(t−Tb)· 4A(j2π)n−k−1fin−k∑m=−∞∞(2m+1)n−k−1ej2πfi(2m+1)t=4A(j2π)n−1finR(t)∑m=−∞∞(2m+1)n−1ej2πfi(2m+1)t+4A∑k=1nnk(j2π)n−k−1fin−k·∑m=−∞∞(2m+1)n−k−1δ(k−1)(t)−δ(k−1)(t−Tb)ej2πfi(2m+1)t.

Upon applying the product rule for the Dirac delta function, i.e., δ(n)(t)f(t)=∑k=0n(−1)knkf(k)(0)δ(n−k)(t), the term $\left(\delta^{\left(k-1\right)}\left(t\right)-\delta^{\left(k-1\right)}\left(t-T_{b}\right)\right)e^{j2\pi f_{i}\left(2m+1\right)t}$ in (19) simplifies to

(20) δ(k−1)(t)−δ(k−1)(t−Tb)ej2πfi(2m+1)t=∑u=0k−1(−1)uk−1uj2πfi(2m+1)uδ(k−1−u)(t)−δ(k−1−u)(t−Tb).

Therefore, $f^{\left(n\right)}\left(t\right)$ can be expressed as

(21) f(n)(t)=4A(j2π)n−1finR(t)∑m=−∞∞(2m+1)n−1ej2πfi(2m+1)t+4A∑k=1nnk(j2π)n−k−1fin−k∑m=−∞∞(2m+1)n−k−1·∑u=0k−1k−1u−j2πfi(2m+1)uδ(k−1−u)(t)−δ(k−1−u)(t−Tb).

The first term in (21) reveals that the spectral amplitude at odd multiples of $f_{i}$ is scaled by $4A{\left(j2\pi \left(2m+1\right)\right)}^{n-1}f_{i}^{n}$, indicating that high-order differentiation amplifies the backscatter signal components at these frequencies by a power-law factor. For the last term in (21), its boundary components are proportional to $\left(\delta^{\left(k-1\right)}\left(t\right)-\delta^{\left(k-1\right)}\left(t-T_{b}\right)\right)$. Taking the Fourier transform yields $F\left\{\delta^{\left(k\right)}\left(t\right)-\delta^{\left(k\right)}\left(t-T_{b}\right)\right\}={\left(j2\pi f\right)}^{k}\left(1-e^{-j2\pi fT_{b}}\right)$. Since $T_{b}=N_{i}T_{i}$ and $f_{i}=1/T_{i}$, it follows that $f_{i}T_{b}=N_{i}$, which is an integer. Consequently, $1-e^{-j2\pi f_{i}T_{b}}=0$, and more generally, the spectral contribution of all boundary impulse terms vanishes at $f=mf_{i}$ for any integer *m*. Hence, the last term in (21) does not interfere with the backscatter signal at the detection frequency $f_{i}$.

### 3.2. Phase-Preserving Signal Detection Method

Based on the above analysis of high-order differentiation, the following division operation is applied, given by

(22) $$\Delta y\left(t\right)=\frac{y^{\left(n\right)}\left(t\right)}{y(t)}.$$

This division serves to normalize the received signal, effectively removing the unknown ambient carrier envelope and emphasizing the relative frequency-shift information carried by the modulation waveform. In practical implementations, a small regularization constant can be added to the denominator to avoid numerical instability when the instantaneous amplitude of y(t) is extremely low, without affecting the detection performance at the target frequencies.

After removing the DC component of Δy(t), the Fourier transform is taken, yielding

(23) $$\Delta Y\left(f\right)=F\left\{\Delta y(t)-E\{\Delta y(t)\}\right\},$$

where F{·} denotes the Fourier transform operator, and the DC term E{Δy(t)} is the time average over one bit period $T_{b}$, i.e., $E\left\{\Delta y\left(t\right)\right\}=\frac{1}{T_{b}}\int_{0}^{T_{b}}\Delta y\left(t\right)\;dt$.

Finally, the information bits are detected by comparing the magnitudes of the relevant frequency components. It is worth clarifying the relationship between the modulation frequency $f_{i}$ analyzed in Section 3.1 and the evaluation frequency $f_{i}/2$ used in the frequency amplitude comparison detection (FACD) criterion. In Section 3.1, the spectrum of the modulation waveform f(t) consists of odd harmonics at $\left(2m+1\right)f_{i}$. After the division operation, the dominant term becomes approximately $f^{\left(n\right)}\left(t\right)$, whose spectrum exhibits peaks at half of the original harmonic frequencies, i.e., at $\left(2m+1\right)f_{i}/2$. Since the fundamental component at $f_{i}/2$ carries most of the signal energy, the FACD rule evaluates only this component, while the higher harmonics are omitted due to their lower energy and higher noise sensitivity. Specifically, if ${\left|\Delta Y\left(\frac{f_{1}}{2}\right)+\Delta Y^{*}\left(-\frac{f_{1}}{2}\right)\right|}^{2}>{\left|\Delta Y\left(\frac{f_{0}}{2}\right)+\Delta Y^{*}\left(-\frac{f_{0}}{2}\right)\right|}^{2}$, the bit is decided as “1”; otherwise, it is decided as “0”. This FACD criterion is formulated as

(24) $$\left\{\begin{array}{cc} {\left|\Delta Y\left(\frac{f_{1}}{2}\right)+\Delta Y^{*}\left(-\frac{f_{1}}{2}\right)\right|}^{2}>{\left|\Delta Y\left(\frac{f_{0}}{2}\right)+\Delta Y^{*}\left(-\frac{f_{0}}{2}\right)\right|}^{2}, & “1”,\\ {\left|\Delta Y\left(\frac{f_{1}}{2}\right)+\Delta Y^{*}\left(-\frac{f_{1}}{2}\right)\right|}^{2}\leq {\left|\Delta Y\left(\frac{f_{0}}{2}\right)+\Delta Y^{*}\left(-\frac{f_{0}}{2}\right)\right|}^{2}, & “0”, \end{array}\right.$$

where $\Delta Y^{*}$ denotes the complex conjugate of ΔY.

The key of the FACD rule lies in its phase-preserving coherent combination of the positive and negative frequency components. For a real-valued signal, its Fourier transform satisfies the conjugate symmetry $\Delta Y\left(-f\right)=\Delta Y^{*}\left(f\right)$. Hence, the term $\Delta Y\left(f/2\right)+\Delta Y^{*}\left(-f/2\right)$ effectively adds the two conjugate-symmetric spectral replicas in phase, yielding a constructive superposition whose magnitude is doubled compared with each individual component. By contrast, a conventional phase-free magnitude comparison would compute ${\left|\Delta Y\left(f/2\right)\right|}^{2}+{\left|\Delta Y\left(-f/2\right)\right|}^{2}$, which is equivalent to incoherent energy summation and does not exploit the phase alignment.

## 4. Performance Analysis

High-order differentiation substantially amplifies the ambient RF signal, the direct-link interference, and the channel noise, which may seriously degrade the detection performance. To quantify these effects, this section analyzes the impact of the derivative order *n* on the average powers of the interference and noise components, and also provides the computational complexity of the proposed detection method.

Throughout this section, we adopt the baseband-equivalent complex signal model, i.e., x(t) is the complex envelope of the ambient RF signal with zero mean and power ${E\{|x\left(t\right)|}^{2}\}=\sigma_{x}^{2}$. Its PSD is $S_{x}\left(\omega \right)$, where ω is the normalized angular frequency with respect to the sampling rate $f_{s}$. The signal bandwidth is *B* (in Hz), and we define $\omega_{B}≜2\pi B/f_{s}\ll 1$. The switching frequency $f_{i}$ of f(t) satisfies $f_{i}\ll f_{s}$ and is of the same order of magnitude as the signal bandwidth *B*.

According to (2), the interference term (excluding the desired signal) and the noise term after *n*-th order differentiation are, respectively,

(25) I(n)(t)=x(n)(t)+∑k=1nnkf(n−k)(t)x(k)(t),

and

(26) w(n)(t)=∑k=0nnkf(n−k)(t)w11(k)(t)+w0(n)(t)+w12(n)(t),

where $w_{11}$, $w_{0}$, and $w_{12}$ are mutually independent band-limited white noise processes.

### 4.1. Power of $I^{\left(n\right)}\left(t\right)$

We define the average power of the interference derivative as

(27) $$P_{I,n}≜E\left\{{\left|I^{\left(n\right)}\left(t\right)\right|}^{2}\right\}.$$

Let $a_{0}=x^{\left(n\right)}\left(t\right)$, and for k≥1, ak=nkf(n−k)(t)x(k)(t). Then $I^{\left(n\right)}\left(t\right)=\sum_{k=0}^{n}a_{k}\left(t\right)$, and

(28) $$P_{I,n}=\sum_{k=0}^{n}\sum_{l=0}^{n}E\left\{a_{k}\left(t\right)a_{l}^{*}\left(t\right)\right\}.$$

Define

(29) $$M_{k,l}≜E\left\{x^{\left(k\right)}\left(t\right){\left(x^{\left(l\right)}\left(t\right)\right)}^{*}\right\},$$

(30) $$N_{p,q}≜E\left\{f^{\left(p\right)}\left(t\right)f^{\left(q\right)}\left(t\right)\right\}.$$

Since x(t) and f(t) are statistically independent and f(t) is real-valued, all terms in (28) are expressed as follows.

- For k=0 and l=0,
  (31) $$E\left\{a_{0}a_{0}^{*}\right\}=E\{|x^{\left(n\right)}\left(t\right){|}^{2}\}≜M_{n,n}.$$
- For k≥1 and l≥1,
  (32) E{akal∗}=nknlE{f(n−k)(t)f(n−l)(t)}E{x(k)(t)(x(l)(t))∗}.
- For k=0 and l≥1 (and symmetrically for k≥1, l=0),

(33) E{a0al∗}=nlE{x(n)(t)(x(l)(t))∗}E{f(n−l)(t)}.

For the zero-mean f(t), all its derivatives have zero mean due to the symmetry of the square wave. All cross terms in (33) vanish. Consequently, $P_{I,n}$ is rewritten as

(34) PI,n=Mn,n+∑k=1n∑l=1nnknlNn−k,n−lMk,l.

For stationary signals, $M_{k,l}={\left(-1\right)}^{l}R_{x}^{\left(k+l\right)}\left(0\right)$, where $R_{x}\left(\tau \right)=E\left\{x\left(t+\tau \right)x^{*}\left(t\right)\right\}$. Hence $M_{k,l}=0$ when k+l is odd; it is nonzero only for even k+l. Similarly, $N_{a,b}$ is nonzero only when a+b is even. In (34), a=n−k and b=n−l, so a+b=2n−(k+l) has the same parity as k+l. Thus, the nonzero terms in (34) have even k+l.

$M_{k,k}$ can be expressed in the frequency-domain form as

(35) $$M_{k,k}≜E\{|x^{\left(k\right)}{\left(t\right)|}^{2}\}=\frac{1}{2\pi }\int_{-\pi }^{\pi }{\left|H_{k}\left(\omega \right)\right|}^{2}S_{x}\left(\omega \right)\;d\omega .$$

Since the signal is bandlimited with $\omega_{B}\ll 1$, the frequency response of the *k*-th derivative is $H_{k}\left(\omega \right)\approx {\left(j\omega \right)}^{k}$, and thus, $|H_{k}{\left(\omega \right)|}^{2}\approx \omega^{2k}$. Consequently, $M_{k,k}$ is approximated as

(36) $$M_{k,k}\approx \frac{1}{2\pi }\int_{-\omega_{B}}^{\omega_{B}}\omega^{2k}S_{x}\left(\omega \right)\;d\omega .$$

If $S_{x}\left(\omega \right)$ is nearly flat over the band *B*, then $M_{k,k}\propto \omega_{B}^{2k+1}$. Therefore,

(37) $$M_{1,1}\gg M_{2,2}\gg M_{3,3}\gg \cdots \gg M_{n,n}.$$

For n≥2, the term corresponding to k=l=1 in the double sum of (34) is

(38) n12Nn−1,n−1M1,1=n2Nn−1,n−1M1,1.

To analyze other terms in the double sum of (34), $R_{x}^{\left(m\right)}\left(0\right)$ is first expressed as

(39) $$R_{x}^{\left(m\right)}\left(0\right)=\frac{1}{2\pi }\int_{-\pi }^{\pi }{\left(j\omega \right)}^{m}S_{x}\left(\omega \right)\;d\omega .$$

Because $S_{x}\left(\omega \right)$ is symmetric, $R_{x}^{\left(m\right)}\left(0\right)$ is zero for odd *m*, while for even m=2p, its magnitude satisfies

(40) $$|R_{x}^{\left(2p\right)}\left(0\right)|\approx \frac{1}{2\pi }\int_{-\omega_{B}}^{\omega_{B}}\omega^{2p}S_{x}\left(\omega \right)\;d\omega \propto \omega_{B}^{2p+1}.$$

Since $M_{k,l}$ is nonzero only when k+l is even, when k+l=2p, we have

(41) $$|M_{k,l}\left|=\right|R_{x}^{\left(2p\right)}\left(0\right)|\propto \omega_{B}^{2p+1}.$$

The smallest even sum (apart from 0) is k+l=2, which occurs only for k=l=1. For any other pair with k+l≥4, we have p≥2, and thus

(42) $$|M_{k,l}|\propto \omega_{B}^{5}\;\mathrm{or}\;\mathrm{smaller}.$$

Since $|M_{1,1}|\propto \omega_{B}^{3}$ and $\omega_{B}\ll 1$, we have $\omega_{B}^{3}\gg \omega_{B}^{5}$. Therefore, any term involving $M_{k,l}$ with k+l≥4 is at least a factor of $\omega_{B}^{2}$ smaller than the k=l=1 term. Moreover, the term with k=l=n yields $M_{n,n}\propto \omega_{B}^{2n+1}$, which is even smaller for n≥2. Although the binomial coefficients may be large, they grow at most polynomially in *n*, whereas the frequency-dependent decay is exponential in k+l since $\omega_{B}<1$. Hence, this exponential decay dominates any polynomial growth of the binomial coefficients, and the k=l=1 term dominates all other terms for n≥2. Accordingly, the interference power can be tightly approximated as

(43) $$P_{I,n}\approx n^{2}N_{n-1,n-1}M_{1,1}.$$

For the square wave f(t), its discontinuities lead to impulsive derivatives. The energy of the *m*-th derivative, $N_{m,m}≜E\{|f^{\left(m\right)}\left(t\right){|}^{2}\}$, is proportional to $A^{2}$ times the sum of squares of binomial coefficients, given by

(44) Nm,m∝A2∑j=0mmj2=A22mm.

Using Stirling’s approximation, 2mm∼4mπm, which grows exponentially with *m*. Hence,

(45) $$N_{1,1}\ll N_{2,2}\ll N_{3,3}\ll \cdots \ll N_{m,m}\ll \cdots .$$

We now compare $P_{I,1}$, $P_{I,2}$, and $P_{I,n}$ for n≥3. The explicit expressions for n=1,2,3 are

(46) PI,1=M1,1+112N0,0M1,1=(1+A2)M1,1,

(47) PI,2=M2,2+212N1,1M1,1+222N0,0M2,2=(1+A2)M2,2+4N1,1M1,1,

(48) PI,3=M3,3+312N2,2M1,1+322N1,1M2,2+332N0,0M3,3=(1+A2)M3,3+9N2,2M1,1+9N1,1M2,2.

From (46) and (47), using $M_{1,1}\gg M_{2,2}$ and $4N_{1,1}\ll 1+A^{2}$ (since $N_{1,1}\ll 1$), we get $P_{I,2}<P_{I,1}$. Also, from (43), (45), and (48), we have $P_{I,2}\approx 4\;N_{1,1}\;M_{1,1}\ll P_{I,3}\approx 9\;N_{2,2}\;M_{1,1}$. For n≥3, (43) together with the monotonic increase in both $n^{2}$ and $N_{n-1,n-1}$ implies $P_{I,3}<P_{I,4}<P_{I,5}<\cdots$. Finally, we conclude that

(49) $$P_{I,2}<P_{I,n}\;\mathrm{for}\;\mathrm{all}\;n\neq 2,\;n\geq 0.$$

Thus, the interference power attains its global minimum at the second-order derivative.

### 4.2. Power of $w^{\left(n\right)}\left(t\right)$

Let

(50) $$P_{w,n}≜E\left\{{\left|w^{\left(n\right)}\left(t\right)\right|}^{2}\right\}.$$

Since $w_{11}\left(t\right)$, $w_{0}\left(t\right)$, and $w_{12}\left(t\right)$ are mutually independent and also independent of f(t), (26) yields

(51) Pw,n=Ew0(n)(t)+w12(n)(t)2+∑k=0nnk2Ef(n−k)(t)2Ew11(k)(t)2.

For a stationary random process z(t) with PSD $S_{z}\left(f\right)$, the power of its *m*-th derivative is $E\left\{{\left|z^{\left(m\right)}\left(t\right)\right|}^{2}\right\}=\int_{-\infty }^{\infty }{\left(2\pi f\right)}^{2m}S_{z}\left(f\right)\;df$. For band-limited white noise with bandwidth *B*, $S_{z}\left(f\right)$ is constant over its support, yielding

(52) $$E\left\{{\left|z^{\left(m\right)}\left(t\right)\right|}^{2}\right\}\propto \int_{-B}^{B}{\left(2\pi f\right)}^{2m}\;df=\frac{2{\left(2\pi \right)}^{2m}B^{2m+1}}{2m+1},$$

which increases monotonically with *m*.

The first term in (51) can be written as

(53) $$E\left\{{\left|w_{0}^{\left(n\right)}+w_{12}^{\left(n\right)}\right|}^{2}\right\}=E\left\{|w_{0}^{\left(n\right)}{|}^{2}\right\}+E\left\{|w_{12}^{\left(n\right)}{|}^{2}\right\},$$

and both terms increase with *n* according to (52).

In the second term of (51), for each *k*, both nk2 and $N_{n-k,n-k}$ (which grows exponentially in n−k) increase with *n*, while $E\{|w_{11}^{\left(k\right)}{|}^{2}\}$ is positive. Therefore, the entire summation also increases with *n*.

Consequently, every component of $P_{w,n}$ in (51) is an increasing function of *n*, which implies

(54) $$P_{w,0}<P_{w,1}<P_{w,2}<P_{w,3}<\cdots ,$$

i.e., the noise power after differentiation is strictly increasing with *n*.

**Figure 4:**
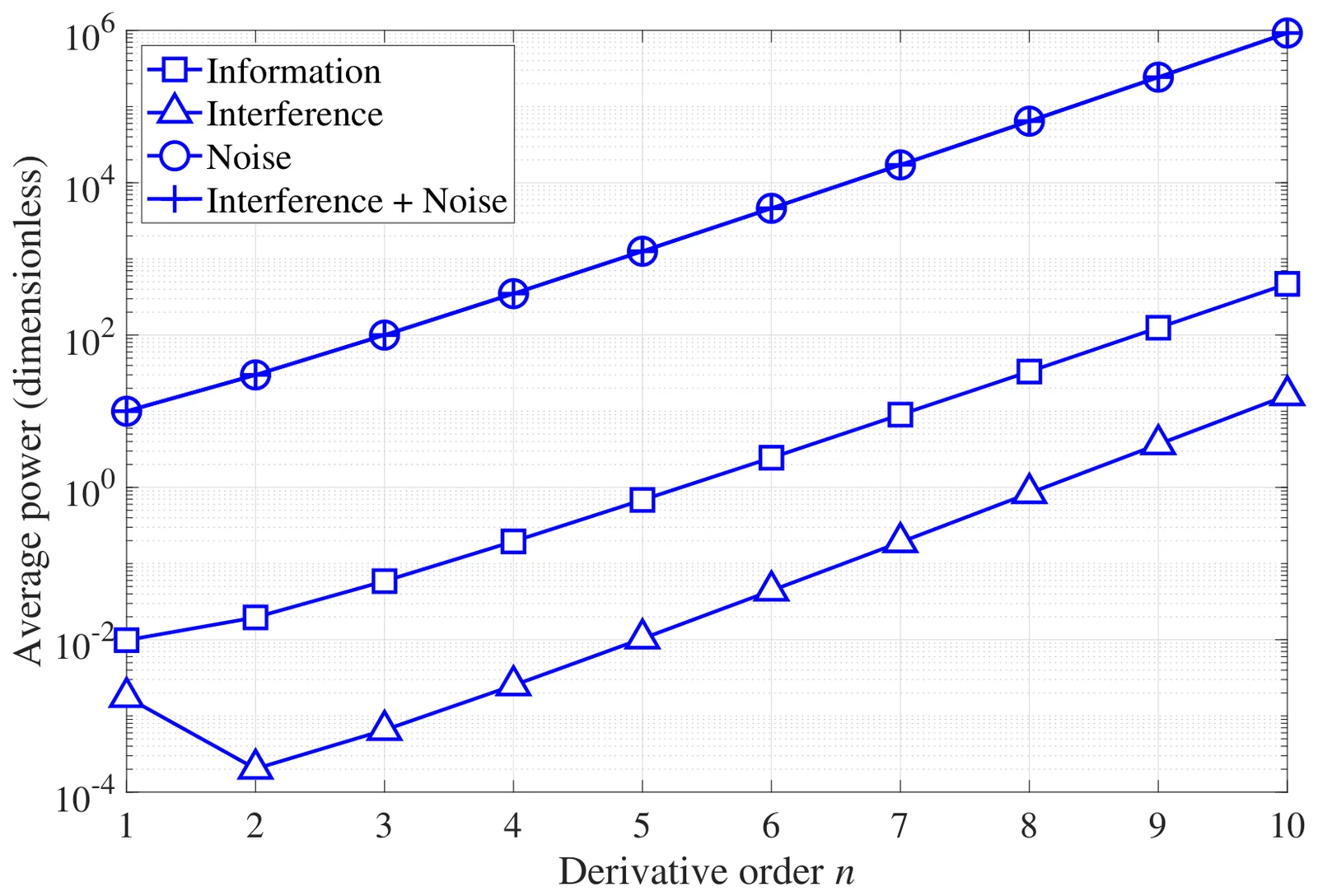
Average power of the information signal $f^{\left(n\right)}\left(t\right)x\left(t\right)$, interference $I^{\left(n\right)}\left(t\right)$, and noise $w^{\left(n\right)}\left(t\right)$ versus derivative order *n*. The vertical axis denotes the average power relative to the direct-path signal x(t), with $E\{|x\left(t\right){|}^{2}\}=1$ as the reference. The large values at higher orders arise from the frequency-domain scaling factor ${\left(j2\pi f\right)}^{n}$ introduced by the *n*th-order derivative.

To validate the analyses in Section 4.1 and Section 4.2, Figure 4 compares the average power of the desired signal $f^{\left(n\right)}\left(t\right)x\left(t\right)$, the interference $I^{\left(n\right)}\left(t\right)$, and the channel noise $w^{\left(n\right)}\left(t\right)$ as functions of the derivative order *n*, with Q=6 dB, SNR =10 dB, and the remaining simulation parameters specified in Section 5. It can be observed that the average powers of all three components grow rapidly with *n*. Notably, the interference power is minimized and the noise power is kept relatively low at n=2, implying the highest signal-to-interference-plus-noise ratio (SINR) and thus the lowest BER among the tested orders.

### 4.3. Complexity Analysis

In practice, signal detection is performed in the discrete-time domain, and the high-order derivative in (2) is approximated by finite differences. From Section 2, $N_{0}$ and $N_{1}$ are the numbers of discrete-time samples for bits “0” and “1”, respectively. To simplify the exposition, we assume that the two information bits occur with equal probability, which yields an average sample count of $L=\frac{N_{0}+N_{1}}{2}$. After applying an *n*th-order finite difference, the sequence length reduces to L−n. The *n*th-order derivative is obtained through *n* successive first-order differences. For an input sequence of length *L*, this requires $\left(L-1\right)+\left(L-2\right)+\cdots +\left(L-n\right)=\frac{n(2L-n-1)}{2}$ complex additions.

The division in (22) is performed on the remaining L−n samples, requiring L−n complex multiplications.

In (23), removing the DC component involves summing L−n samples (requiring L−n−1 complex additions) and then subtracting the mean from each sample (requiring L−n complex additions), totaling 2(L−n)−1 complex additions. The subsequent fast Fourier transform (FFT) of length L−n contributes $\frac{L-n}{2}\log_{2}\left(L-n\right)$ complex multiplications and $\left(L-n\right)\log_{2}\left(L-n\right)$ complex additions.

Finally, the frequency amplitude comparison in (24) requires two complex additions and two complex multiplications for magnitude squares, and one amplitude comparison.

Combining all the above contributions, the total numbers of complex multiplications $C_{\times }$ and complex additions $C_{+}$ are

(55) $$C_{\times }=\left(L-n\right)+\frac{L-n}{2}\log_{2}\left(L-n\right)+2,$$

(56) $$C_{+}=\frac{n(2L-n-1)}{2}+2\left(L-n\right)+\left(L-n\right)\log_{2}\left(L-n\right)+1.$$

The complexity is dominated by the FFT term.

Since complex additions and multiplications are of the same order of magnitude, Table 1 compares the dominant complex multiplications and the number of amplitude comparisons required per symbol decision for the detection schemes introduced in Section 5. As shown, the Max-based detectors require $O(M^{2})$ complex multiplications and *M* amplitude comparisons, where M=L−n, because they evaluate all candidate frequency bins. In contrast, the FFT-based FACD variants reduce the computational complexity to O(MlogM) while requiring only a single amplitude comparison. The proposed phase-preserving FACD achieves the same order of complexity as the phase-free FACD, yet delivers the best BER performance among all compared methods, as will be demonstrated in Section 5.

## 5. Simulation Results

In our simulation, the baseband-equivalent ambient RF source employs quadrature phase-shift keying (QPSK) modulation with a sampling frequency of 2 MHz and a bandwidth of 40 kHz. The signal is oversampled by a factor of 50 using a square-root raised-cosine FIR filter with a roll-off factor of 0.25. The information bit rate $R_{b}$ of the BD is set to 200 bps. FSK modulation is implemented with a square wave, where frequencies $f_{0}=16$ kHz and $f_{1}=20$ kHz correspond to bits “0” and “1”, respectively. Following the system model in Section 2, the SNR is defined as the power ratio of the RF source signal to the total channel noise from both the direct and backscatter paths, i.e., ${\mathrm{SNR}}_{D}=10\log_{10}\frac{\frac{1}{T_{b}}\int_{0}^{T_{b}}{\left|x\left(t\right)\right|}^{2}\;dt}{\frac{1}{T_{b}}\int_{0}^{T_{b}}{\left|w\left(t\right)\right|}^{2}\;dt}$. The power ratio of the FSK-modulated signal in the backscatter path to the RF source signal in the direct path is defined as

(57) $$Q=-10\log_{10}\frac{\frac{1}{T_{b}}\int_{0}^{T_{b}}{\left|f\left(t\right)x\left(t\right)\right|}^{2}\;dt}{\frac{1}{T_{b}}\int_{0}^{T_{b}}{\left|x\left(t\right)\right|}^{2}\;dt}=-20\log_{10}A\;\mathrm{dB}.$$

For fair comparison, the following baseline methods are considered.

- Max (phase-free): According to [28], from the frequency-domain signal ΔY(f), the frequency with the maximum spectral amplitude is identified as
  (58) $$f^{*}=\arg\max_{f\in B}\left\{{\left|\Delta Y(f)\right|}^{2}\right\},$$
  where $B=\{b_{1},b_{2},\ldots ,b_{N_{B}}\}$ denotes the set of frequency bins associated with the backscatter signal, with $b_{1}$ and $b_{N_{B}}$ being the start and end frequencies, and $N_{B}=\left|B\right|$. The estimated information bit is then obtained by comparing $f^{*}$ with the FSK modulation frequencies.
- Max (phase-preserve): To exploit phase information, the conjugate symmetry of ΔY(f) is used, i.e.,
  (59) $$f^{*}=\arg\max_{\hat{f}\in \hat{B},\bar{f}\in \bar{B}}\left\{{\left|\Delta Y\left(\hat{f}\right)+\Delta Y\left(\bar{f}\right)\right|}^{2}\right\},$$
  where $\bar{B}=\left\{b_{N_{B}/2+1},b_{N_{B}/2+2},\ldots ,b_{N_{B}}\right\}$ denotes the corresponding negative-frequency bins (in the discrete Fourier transform, these are represented by the upper half of the spectrum), $\hat{B}=\left\{b_{1},b_{2},\ldots ,b_{N_{B}/2}\right\}$ denotes the positive-frequency bins, $\hat{B}\cup \bar{B}=B$, and $\hat{B}\cap \bar{B}=\emptyset$. The decision on the information bit follows the same frequency comparison procedure as above.
- FACD (phase-free): Unlike (24), this method adopts a phase-free detection criterion, expressed as
  (60) $$\left\{\begin{array}{cc} {\left|\Delta Y\left(\frac{f_{1}}{2}\right)\right|}^{2}+{\left|\Delta Y^{*}\left(-\frac{f_{1}}{2}\right)\right|}^{2}>{\left|\Delta Y\left(\frac{f_{0}}{2}\right)\right|}^{2}+{\left|\Delta Y^{*}\left(-\frac{f_{0}}{2}\right)\right|}^{2}, & “1”,\\ {\left|\Delta Y\left(\frac{f_{1}}{2}\right)\right|}^{2}+{\left|\Delta Y^{*}\left(-\frac{f_{1}}{2}\right)\right|}^{2}\leq {\left|\Delta Y\left(\frac{f_{0}}{2}\right)\right|}^{2}+{\left|\Delta Y^{*}\left(-\frac{f_{0}}{2}\right)\right|}^{2}, & “0”. \end{array}\right.$$

**Figure 5:**
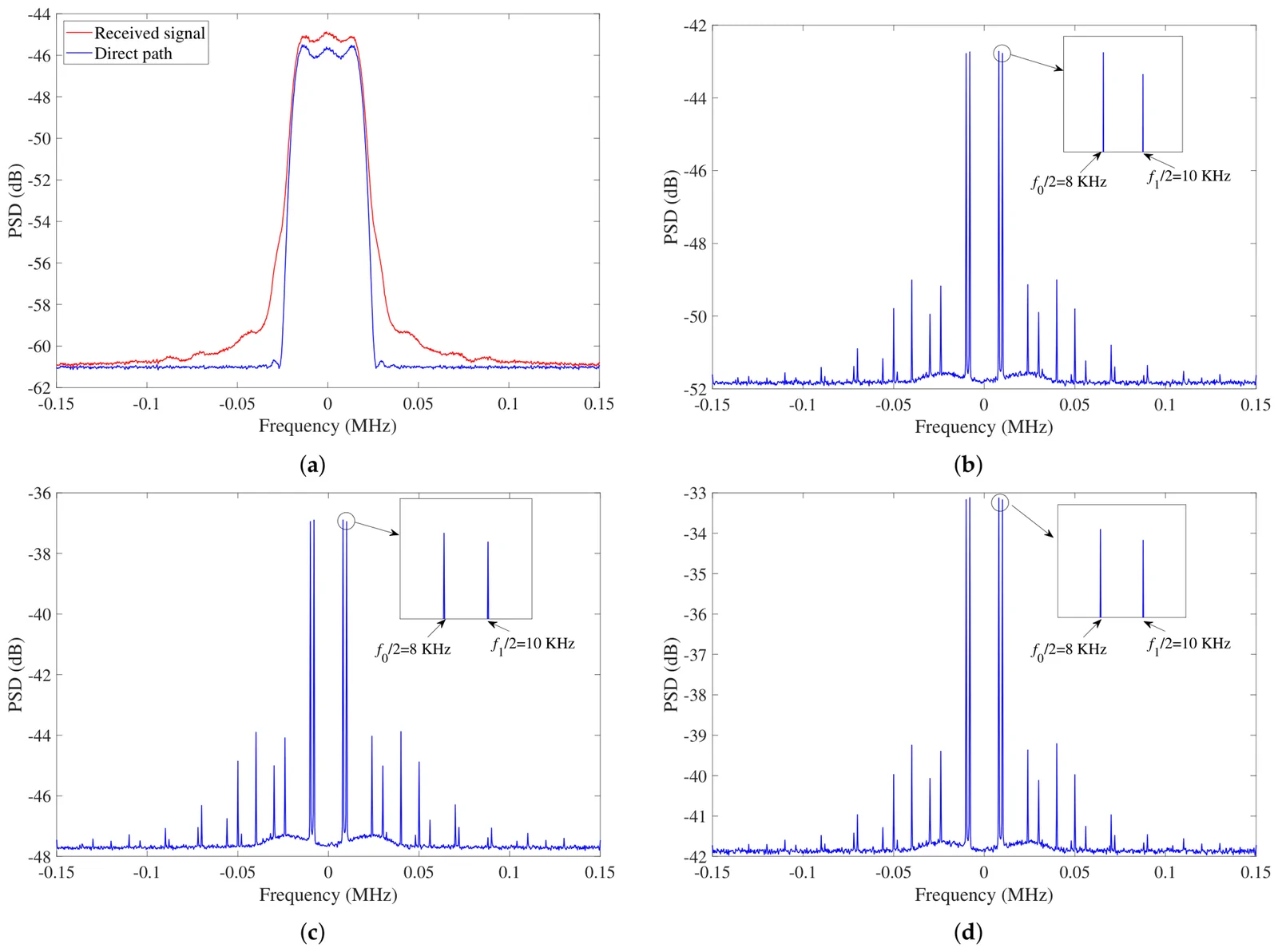
PSD of the received signal and its derivatives: (**a**) Original received signal (n=0). (**b**) First-order derivative (n=1). (**c**) Second-order derivative (n=2). (**d**) Third-order derivative (n=3).

Figure 5 compares the PSDs of the received signal and its higher-order derivatives for ${\mathrm{SNR}}_{D}=15$ dB and Q=6 dB. Figure 5a shows the PSD of the signal received via the direct path as a reference. Figure 5b–d correspond to n=1,2,3, respectively, where their DC components are removed. As observed from Figure 5a, the backscatter signal has only a minor effect within the RF source band, while a moderate spectral growth appears just outside that band. Figure 5b–d reveal that the spectral amplitude increases with the derivative order *n*. Moreover, consistent with the results in Figure 4, the spectral separation between the modulation frequency $f_{i}$ and the channel noise is maximized at n=2.

**Figure 6:**
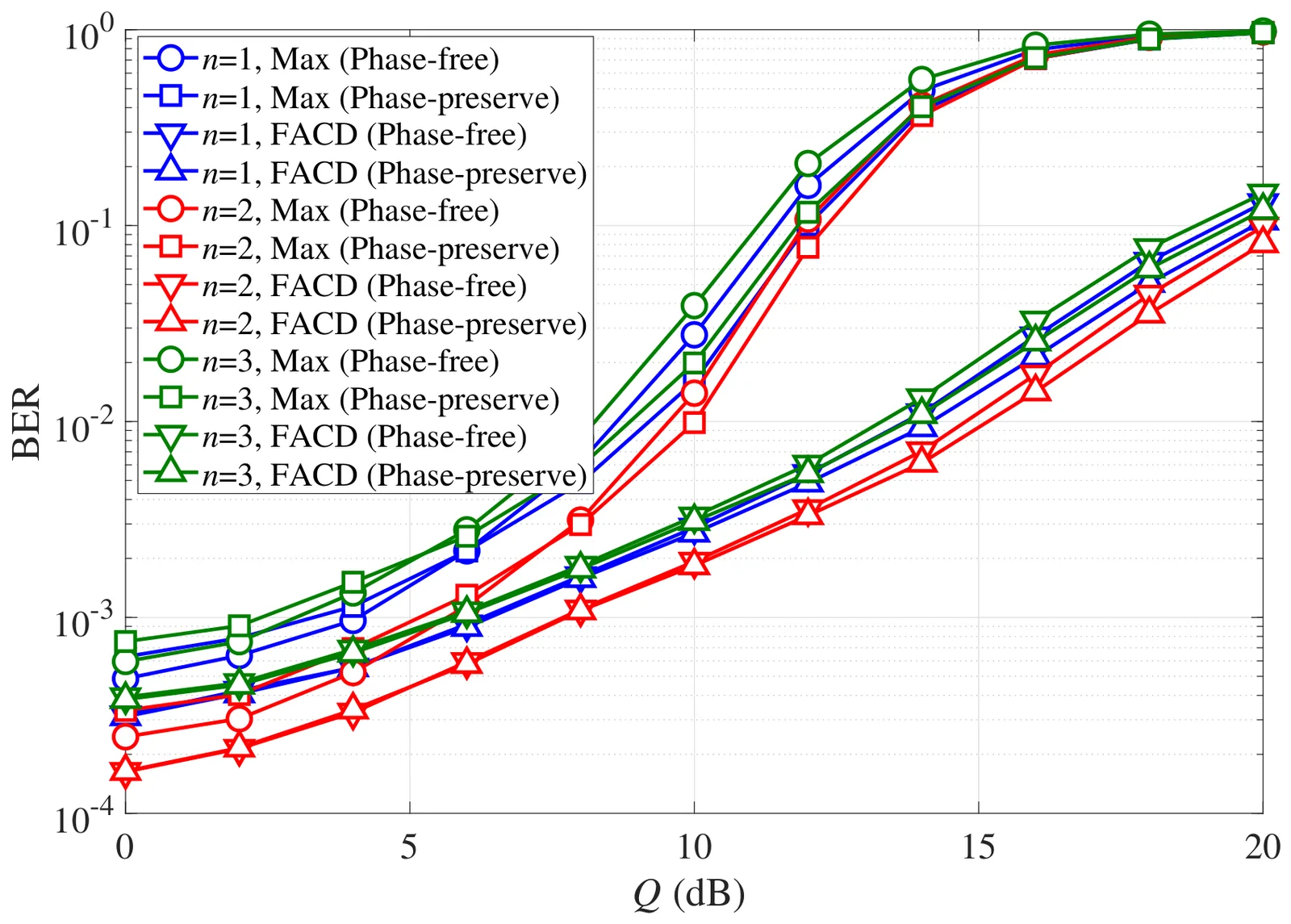
BER versus *Q* for the FSK-modulated AmBC signals.

Figure 6 compares the BER performance of Max (phase-free), Max (phase-preserve), FACD (phase-free), and the proposed FACD (phase-preserve) for different derivative orders. Here, ${\mathrm{SNR}}_{D}=20$ dB, and *Q* varies from 0 to 20 dB. It is evident that the proposed FACD (phase-preserve) with n=2 achieves the lowest BER among all tested schemes, outperforming both the same method with n=1 or n=3 and the baseline methods. Furthermore, as *Q* increases, a significant degradation in error performance is observed, owing to the reduced power of the backscatter signal.

**Figure 7:**
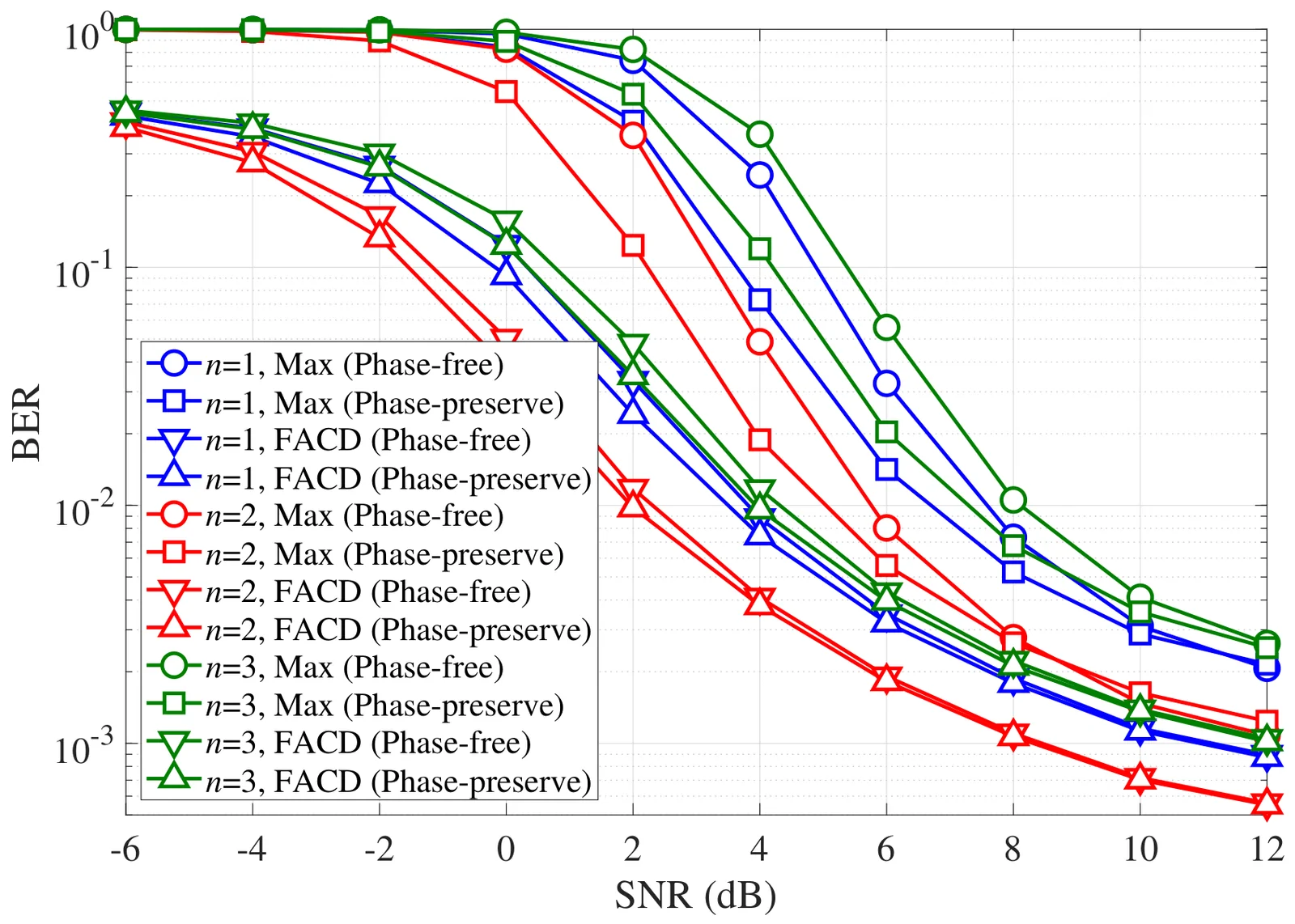
BER versus SNR for the FSK-modulated AmBC signals with the default parameter set ($f_{s}=2$ MHz, B=40 kHz, $R_{b}=200$ bps, $f_{0}=16$ kHz, $f_{1}=20$ kHz).

Furthermore, Figure 7 examines the BERs of the baseline methods and the proposed FACD (phase-preserve) with varying received SNRs. The received SNR is defined as the ratio of the total received signal power to the total noise power, i.e., ${\mathrm{SNR}}_{B}=10\log_{10}\frac{\frac{1}{T_{b}}\int_{0}^{T_{b}}{\left|x(t)+f(t)x(t)\right|}^{2}\;dt}{\frac{1}{T_{b}}\int_{0}^{T_{b}}{\left|w(t)\right|}^{2}\;dt}=10\log_{10}\frac{P_{\mathrm{direct}}+P_{\mathrm{backscatter}}}{P_{\mathrm{noise}}}$. $P_{\mathrm{direct}}$, $P_{\mathrm{backscatter}}$, and $P_{\mathrm{noise}}$ denote the average powers of the direct-path signal, the backscattered signal, and the total channel noise, respectively. Let $P_{\mathrm{backscatter}}=P_{\mathrm{direct}}\cdot 10^{-Q/10}$. The direct-path SNR is ${\mathrm{SNR}}_{D}=10\log_{10}P_{\mathrm{direct}}/P_{\mathrm{noise}}$. Consequently, ${\mathrm{SNR}}_{B}={\mathrm{SNR}}_{D}+10\log_{10}\left(1+10^{-Q/10}\right)$. In this evaluation, we set Q=6 dB and let ${\mathrm{SNR}}_{D}$ range from 0 to 18 dB. Consistent with Figure 6, the proposed FACD (phase-preserve) with n=2 delivers the best error performance. As the SNR increases, the BER improves accordingly. Therefore, for FSK-modulated ambient backscatter signals, the second-order derivative-based detection method offers a better solution compared with baseline methods.

**Figure 8:**
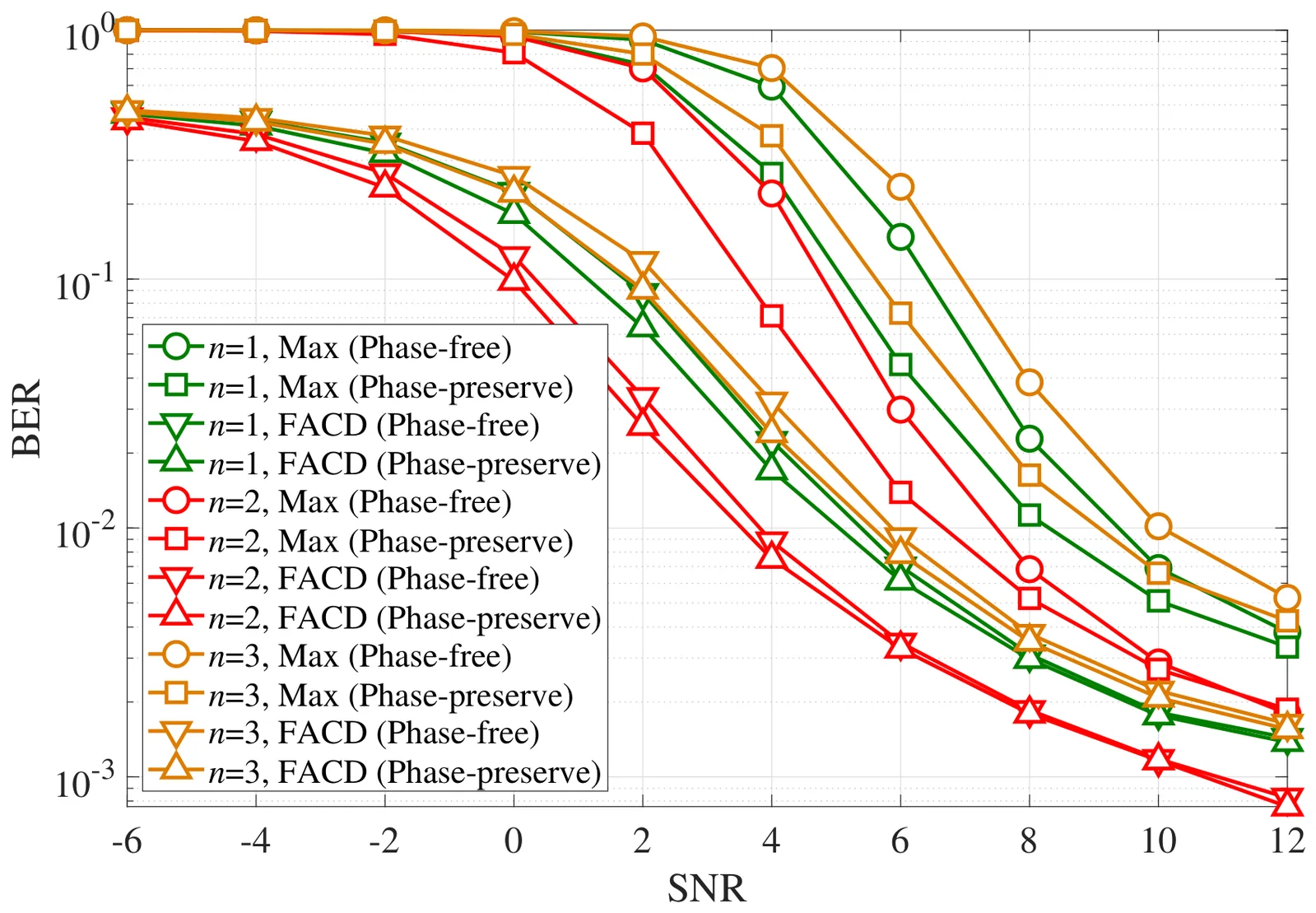
BER versus SNR for the FSK-modulated AmBC signals with an alternative parameter set ($f_{s}=3$ MHz, B=50 kHz, $R_{b}=400$ bps, $f_{0}=8$ kHz, $f_{1}=16$ kHz).

To further validate the robustness of the proposed detector under different parameter settings, Figure 8 presents the BER performance for a sampling rate of 3 MHz, a bandwidth of 50 kHz, a bit rate of 400 bps, and switching frequencies $f_{0}=8$ kHz and $f_{1}=16$ kHz. Consistent with Figure 7, the second-order derivative-based method also achieves the lowest BER among all compared schemes.

## 6. Conclusions

In this paper, a high-order derivative-based signal detection framework was developed for FSK-modulated ambient backscatter communication. Through theoretical analysis, we characterized the power scaling of direct-link interference and channel noise with respect to the derivative order. The results showed that the interference power is minimized at the second order while the noise power increases monotonically with the order, establishing the second-order derivative as the most favorable choice in the considered AmBC regime. A complexity analysis further confirmed that the proposed detector incurs the same order of complexity as FFT-based baselines. Based on this insight, a phase-preserving FACD scheme was proposed. Simulation results confirmed that the proposed second-order FACD method consistently achieves the lowest bit error rate compared with both the first-order and third-order variants as well as conventional max-based detectors, particularly under low-SNR and unfavorable power-ratio conditions.

Although this study assumes AWGN channels, perfect synchronization, and negligible delay to isolate the gain of the second-order derivative, practical deployments would require additional synchronization and equalization modules to cope with multipath fading, frequency offset, and phase noise, as well as regularization of the division operation. A thorough sensitivity analysis of the proposed detector to timing offsets, carrier-frequency offsets, sampling-frequency errors, and imperfect symbol synchronization is also left for future investigation. Nevertheless, the proposed framework provides a clear benchmark and a solid foundation for robust FSK-AmBC receivers in large-scale distributed edge-intelligent sensing. By ensuring reliable data delivery over severely power- and SNR-constrained links, this work also contributes to the trustworthiness of the sensed data acquisition chain. Extending the proposed framework to sensing-oriented metrics, such as estimation error propagation or age of information, constitutes another promising direction for future research.

## Figures and Tables

**Table 1:** Complexity comparison of detection schemes.

Scheme	Number of Complex Multiplications	Number of Amplitude Comparisons
Max (phase-free)	$O\left({\left(L-n\right)}^{2}\right)$	L−n
Max (phase-preserve)	$O\left({\left(L-n\right)}^{2}\right)$	L−n
FACD (phase-free)	$O\left(\frac{L-n}{2}\log_{2}\left(L-n\right)\right)$	1
FACD (phase-preserve)	$O\left(\frac{L-n}{2}\log_{2}\left(L-n\right)\right)$	1

## Data Availability

The original contributions presented in this study are included in the article. Further inquiries can be directed to the corresponding author.

## Abbreviations

The following abbreviations are used in this manuscript:

AmBC	Ambient backscatter communication
AWGN	Additive white Gaussian noise
BD	Backscatter device
BER	Bit error rate
DLI	Direct link interference
FACD	Frequency amplitude comparison detection
FSK	Frequency-shift keying
IoT	Internet of Things
OOK	On–off keying
PSD	Power spectral density
RF	Radio frequency
RFID	Radio-frequency identification
SNR	Signal-to-noise ratio
